# Accuracy and Cost-effectiveness of Technology-Assisted Dietary Assessment Comparing the Automated Self-administered Dietary Assessment Tool, Intake24, and an Image-Assisted Mobile Food Record 24-Hour Recall Relative to Observed Intake: Protocol for a Randomized Crossover Feeding Study

**DOI:** 10.2196/32891

**Published:** 2021-12-16

**Authors:** Clare Whitton, Janelle D Healy, Clare E Collins, Barbara Mullan, Megan E Rollo, Satvinder S Dhaliwal, Richard Norman, Carol J Boushey, Edward J Delp, Fengqing Zhu, Tracy A McCaffrey, Sharon I Kirkpatrick, Paul Atyeo, Syed Aqif Mukhtar, Janine L Wright, César Ramos-García, Christina M Pollard, Deborah A Kerr

**Affiliations:** 1 School of Population Health Faculty of Health Sciences Curtin University Perth Australia; 2 School of Health Sciences College of Health, Medicine and Wellbeing University of Newcastle Newcastle Australia; 3 Priority Research Centre in Physical Activity and Nutrition University of Newcastle Newcastle Australia; 4 Enable Institute Faculty of Health Sciences Curtin University Perth Australia; 5 Curtin Health Innovation Research Institute Faculty of Health Sciences Curtin University Perth Australia; 6 Duke-NUS Medical School National University of Singapore Singapore Singapore; 7 Institute for Research in Molecular Medicine (INFORMM) Universiti Sains Malaysia Pulau Pinang Malaysia; 8 Department of Radiation Oncology Sir Charles Gairdner Hospital Perth Australia; 9 Epidemiology Program University of Hawaii Cancer Center Honolulu, HI United States; 10 School of Electrical and Computer Engineering Purdue University West Lafayette, IN United States; 11 Department of Nutrition, Dietetics and Food Monash University Melbourne Australia; 12 School of Public Health Sciences University of Waterloo Waterloo, ON Canada; 13 Health Section Health and Disability Branch Australian Bureau of Statistics Canberra Australia; 14 Division of Health Sciences Tonalá University Center University of Guadalajara Guadalajara Mexico

**Keywords:** 24-hour recall, Automated Self-Administered Dietary Assessment Tool, Intake24, mobile food record, image-assisted dietary assessment, validation, controlled feeding, accuracy, dietary measurement error, self-report, energy intake, adult, cost-effectiveness, acceptability, mobile technology, diet surveys, mobile phone

## Abstract

**Background:**

The assessment of dietary intake underpins population nutrition surveillance and nutritional epidemiology and is essential to inform effective public health policies and programs. Technological advances in dietary assessment that use images and automated methods have the potential to improve accuracy, respondent burden, and cost; however, they need to be evaluated to inform large-scale use.

**Objective:**

The aim of this study is to compare the accuracy, acceptability, and cost-effectiveness of 3 technology-assisted 24-hour dietary recall (24HR) methods relative to observed intake across 3 meals.

**Methods:**

Using a controlled feeding study design, 24HR data collected using 3 methods will be obtained for comparison with observed intake. A total of 150 healthy adults, aged 18 to 70 years, will be recruited and will complete web-based demographic and psychosocial questionnaires and cognitive tests. Participants will attend a university study center on 3 separate days to consume breakfast, lunch, and dinner, with unobtrusive documentation of the foods and beverages consumed and their amounts. Following each feeding day, participants will complete a 24HR process using 1 of 3 methods: the Automated Self-Administered Dietary Assessment Tool, Intake24, or the Image-Assisted mobile Food Record 24-Hour Recall. The sequence of the 3 methods will be randomized, with each participant exposed to each method approximately 1 week apart. Acceptability and the preferred 24HR method will be assessed using a questionnaire. Estimates of energy, nutrient, and food group intake and portion sizes from each 24HR method will be compared with the observed intake for each day. Linear mixed models will be used, with 24HR method and method order as fixed effects, to assess differences in the 24HR methods. Reporting bias will be assessed by examining the ratios of reported 24HR intake to observed intake. Food and beverage omission and intrusion rates will be calculated, and differences by 24HR method will be assessed using chi-square tests. Psychosocial, demographic, and cognitive factors associated with energy misestimation will be evaluated using chi-square tests and multivariable logistic regression. The financial costs, time costs, and cost-effectiveness of each 24HR method will be assessed and compared using repeated measures analysis of variance tests.

**Results:**

Participant recruitment commenced in March 2021 and is planned to be completed by the end of 2021.

**Conclusions:**

This protocol outlines the methodology of a study that will evaluate the accuracy, acceptability, and cost-effectiveness of 3 technology-enabled dietary assessment methods. This will inform the selection of dietary assessment methods in future studies on nutrition surveillance and epidemiology.

**Trial Registration:**

Australian New Zealand Clinical Trials Registry ACTRN12621000209897; https://tinyurl.com/2p9fpf2s

**International Registered Report Identifier (IRRID):**

DERR1-10.2196/32891

## Introduction

### Background

Dietary intake surveillance enables monitoring of diet-related health and nutritional status of populations and provides vital data to inform public health nutrition policies and programs [1]. Therefore, accurate assessment of dietary intake is important to assist effective government decision-making on dietary advice and programs by defining the extent of the problem and possible solutions. The 24-hour dietary recall (24HR) is the current standard and preferred method for large-scale population surveillance to assess absolute dietary intakes [2-6]. Technology-assisted 24HR offers the potential to improve accuracy and reduce participant and researcher burden. However, the relative accuracy, acceptability, and cost of various technology-assisted methods are unclear. Population dietary surveillance studies typically include thousands of participants. As such, cost and personnel requirements are major determinants of the feasibility of a dietary assessment method. However, information on the cost of method administration has rarely been collected or published. This information would contribute to the feasibility assessment by researchers and decision makers.

A 24HR method is designed to capture detailed information on foods and beverages consumed on the previous day or during the previous 24 hours and is traditionally conducted as a structured face-to-face interview [7]. The 24HR method is a complex process involving numeracy, perception, memory, and the conceptualization of that memory [8]. The benefits of 24HR methods over less burdensome methods, such as food frequency questionnaires, include the detailed accounting of all foods and beverages consumed and information on context (eg, meal timing). This information allows the examination of questions related to different dietary components, dietary patterns, and meal patterning [9].

Various methods have been developed to enhance recall and reduce errors in reported intake. For example, the National Health and Nutrition Examination Survey in the United States includes an interviewer-administered Automated Multiple-Pass Method (AMPM) 24HR [3] as does the Australian Health Survey [2]. AMPM is a web-based interface designed for surveillance, typically implemented in-person by a trained interviewer, which adds to the cost of undertaking large-scale surveys. The AMPM provides a structured interview format with specific probes in 5 structured sets *or passes*: a quick list, forgotten foods pass, time and occasion pass, detail pass, and final review [10]. Portion size estimation is addressed using a food model booklet. More recently, web-based interfaces have been developed to enable self-administration of the 24HR method by participants, removing the need for trained interviewers and reducing study costs.

Automated web-based, self-administered 24HR methods begin with a quick list. Details are collected using a sequence of probes, and standard images of foods or models are used to help participants estimate portion sizes. The format and number of images are informed by user testing [11,12]. These methods include the US-developed Automated Self-Administered Dietary Assessment Tool (ASA24) and the UK-developed Intake24. ASA24 is an adaptation of AMPM and was developed by the United States National Cancer Institute based on input from stakeholders in an external working group, along with cognitive and usability testing [13]. Intake24 was developed by Newcastle University, United Kingdom, using 4 cycles of user testing, with modification after each cycle, in adolescents and young adults [12,14]. Similar levels of measurement error have been observed in web-based self-administered 24HR methods (Intake24 and ASA24) and in interviewer-administered methods as determined by objective measures of energy intake using doubly labeled water [15,16]. This suggests that the additional costs associated with interviewer-administered methods in the form of trained interviewers and coders may not translate to improved accuracy. However, to date, no study has evaluated the differences in accuracy between Intake24 and ASA24.

Using image-assisted methods to supplement the 24HR method has the potential to reduce recall bias, with images used to assist food identification and portion size estimation. In recent reviews, image-assisted approaches, including 24HR methods, resulted in greater accuracy of self-reported dietary intake when compared with the accuracy of methods without images supplied by participants [17-19]. The Image-Assisted mobile Food Record 24-Hour Recall (mFR24) is a mobile app developed by Purdue University. Participants are instructed to take *before* and *after* images of all food and beverages consumed and to include a fiducial marker (an object of known shape, size, and color) [20] in each image to aid in portion size verification. The content of the images is confirmed either by a human trained analyst or by automated methods using computer vision and machine learning (eg, deep learning) techniques [21-23]. In contrast to other image-assisted 24HR methods in which the image review occurs toward or at the end of the interview [24-28], the image review in mFR24 begins at the start of the interview based on participant feedback from pilot testing. This novel approach has yet to be evaluated for its accuracy and acceptability. Furthermore, the accuracy and acceptability of the mFR24 has not been compared with either the web-based self-administered ASA24 or Intake24 in the same study population.

To continue to improve upon dietary assessment, there is an urgent need for studies that enable the understanding and mitigation of measurement errors [29]. The error in dietary intake estimation can be identified by comparing reported intake with recovery biomarkers, such as doubly labeled water as a measure of true energy intake [30]. However, such methods are limited to energy or single nutrients and do not identify specific foods and beverages that are omitted or inaccurately reported. Controlled feeding studies allow for the examination of measurement errors at the level of foods and beverages. Studies with measures of known food and beverage intake enable the understanding of factors contributing to misreporting, such as omission of particular types of foods, intrusions, inaccurate portion size estimation, and incorrect food descriptions [31-35]. For example, Kirkpatrick et al [33] found that more intrusions (items not consumed) were present with the use of a web-based self-administered 24HR method than in an interviewer-administered 24HR method. Widaman et al [35] found no statistically significant differences between estimated and observed intake of grain foods using web-based self-administered 24HR methods, although all other food groups were overestimated. These findings illustrate how controlled feeding studies can provide insights into the mechanisms of dietary intake measurement errors that would otherwise remain unknown.

Various psychosocial factors have been associated with misreporting, including social desirability traits, restraint, disinhibition, fear of negative evaluation, and body weight and body image perceptions [36-38]. In a study conducted in the United States, measuring an array of psychosocial and demographic factors, Tooze et al [37] found that although these factors cumulatively accounted for 20% of the variability in misreporting in interviewer-administered 24HR methods, 80% remained unaccounted for. Clearly, other constructs associated with misreporting, psychosocial or otherwise, need to be identified, and it has been recommended that future research focuses on this [37,39]. For example, it is frequently stated that visual perception and conceptualization of memory are involved in reporting of intake [8], but these factors are not typically assessed in studies evaluating participant reporting accuracy. Furthermore, a better understanding of how psychosocial and cognitive factors map to various sources of error (eg, omissions and intrusion) could potentially help to minimize measurement error. To the best of our knowledge, no controlled feeding studies have evaluated the associations of psychosocial or cognitive factors in food and beverage reporting accuracy.

Social factors may also contribute to misreporting. Individual work patterns and lifestyles have rapidly changed with the increased use of screen-based work and leisure activities [40,41]. In a survey of over 12,000 households in Australia, 75% people reported that they always, often, or sometimes felt rushed or pressed for time [42]. With these rapid shifts in day-to-day life, the demands for traditional dietary assessment methods may be misaligned with people’s daily lives and expectations and may be viewed as inconvenient. Technology-based self-report dietary assessment methods enable remote completion using laptops or mobile devices and have been indicated as more acceptable to participants than traditional face-to-face methods [43-45]. For example, research conducted in the United States demonstrated that 70% preferred the ASA24 over the AMPM [46]. The acceptability of dietary assessment methods has important implications, especially in large-scale population studies, as it can impact response rates and therefore the representativeness and generalizability of the study sample [47,48]. Image-assisted 24HR methods, such as the mFR24, are yet to be fully evaluated for consumer acceptability and comparison with other technology 24HR methods such as Intake24 and ASA24.

### Objectives

This research protocol will compare 3 leading technology-assisted dietary assessment methods. Using a controlled feeding study design with healthy adults aged 18 to 70 years, this study will (1) compare the accuracy, acceptability, and cost-effectiveness of 3 technology-assisted 24HR methods (ASA24, Intake24, and mFR24) relative to observed intake for 3 meals on 1 day; (2) test the accuracy of automated methods for determining food and beverage intake using food images and image analysis, computer vision, and machine learning techniques; and (3) assess associations between reporting errors and demographic, psychosocial, and cognitive factors.

## Methods

### Sample and Recruitment

The sample will be selected from adults aged 18-70 years residing in Perth, the capital city of Western Australia, Australia, and recruited via the electoral roll by selecting postcodes that provide representation across socioeconomic status. Other recruitment methods will include advertising on the Curtin University website and a snowball methodology (eg, email newsletter and referrals from friends or colleagues). A final sample of 150 randomized participants (allowing for a 20% dropout) will allow for 90% power at a 5% significance level when the true difference between any 2 mean differences between estimated and observed dietary energy intake is 0. Quota sampling will be used to ensure that equal numbers of men and women are recruited. To be included in the study, participants must be able to attend in-person feeding sessions on 3 separate days and have access to a computer and a smartphone (running iPhone operating system or Android operating system) with a data plan. Exclusion criteria include serious illnesses or medical conditions, pregnancy, special dietary requirements, or dietary restrictions because of food allergies or intolerances or dieting to lose weight.

### Study Design

**Figure 1:**
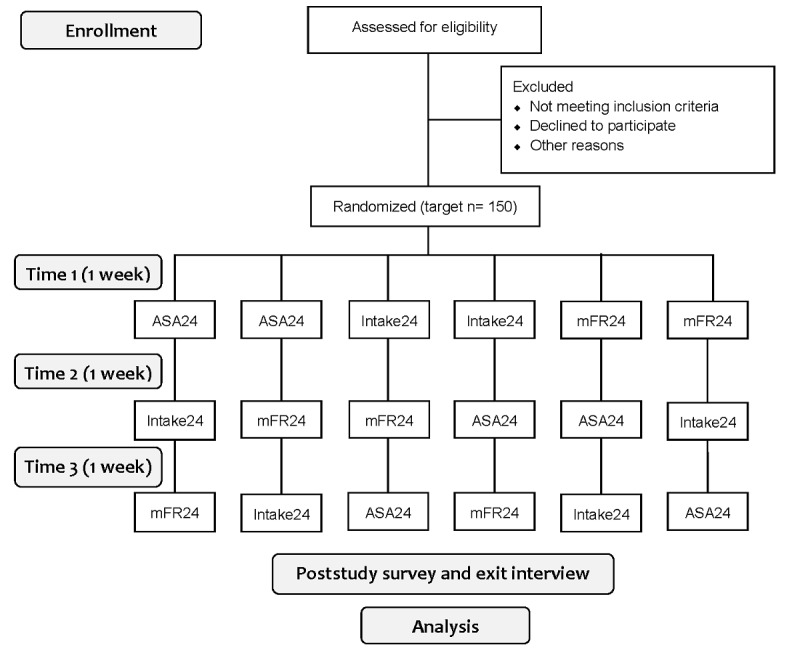
Accuracy, acceptability, and cost-effectiveness of technology-assisted dietary assessment study flowchart on enrollment, randomization, and study design. ASA24: Automated Self-Administered Dietary Assessment Tool; mFR24: Image-Assisted mobile Food Record 24-Hour Recall.

A controlled feeding study with a crossover design will compare the accuracy of 3 technology-assisted methods of assessing 1 day of dietary intake: ASA24, Intake24, and mFR24. The sequence of the 3 dietary assessment periods will be randomized for each participant, with a 1-week washout period between each feeding session (Figure 1). Therefore, each participant will be exposed to each of the 3 methods at different periods. The crossover design, a repeated measurement design, will allow both between-group and within-group method comparisons. This design yields a more efficient comparison of treatments than a parallel design because fewer participants are required in the crossover design to attain the same level of statistical power or precision as a parallel design.

Ethics approval from the Curtin University Human Research Ethics Office has been obtained (approval number: HRE2019-0222). Reciprocal ethics approval from the Department of Health Western Australia Human Research Ethics Committee has also been obtained (approval number: 201909.06), and the trial is registered with the Australian New Zealand Clinical Trials Registry (ACTRN12621000209897). All research design, practices, and reporting of studies conducted in Australia will be aligned with the Australian Code for the Responsible Conduct of Research. Participants will receive a maximum of Aus $60 (US $42) as a token of appreciation for their involvement in the study.

### Research Study Database

A purpose-built research study database will be developed using a database platform (Microsoft Office Professional Plus 2016, Microsoft Pty. Ltd) to manage, contact, and track the progress of the study participants throughout the study. The database will send autogenerated emails containing study information and personalized links to remind participants of upcoming appointments and complete applicable surveys beforehand. Email and SMS text messaging prompts will be sent directly from the study database using *Email to SMS* technology. The study database will automatically update the participant status with respect to their study compliance. The system will prompt reminders via email and SMS text messaging for participants who have not yet completed their tasks.

### Randomization

At the first face-to-face session, eligible participants will be randomized using a random number generator and stratified by gender. The order in which the participant completes the three 24HR methods will be randomly allocated to ensure no order effect. Allocation will be concealed using sealed opaque envelopes. A statistician, not involved in data collection, will generate the randomization sequence. This will ensure adequate allocation concealment from the research team involved in recruitment and data collection.

### Procedures

**Figure 2:**
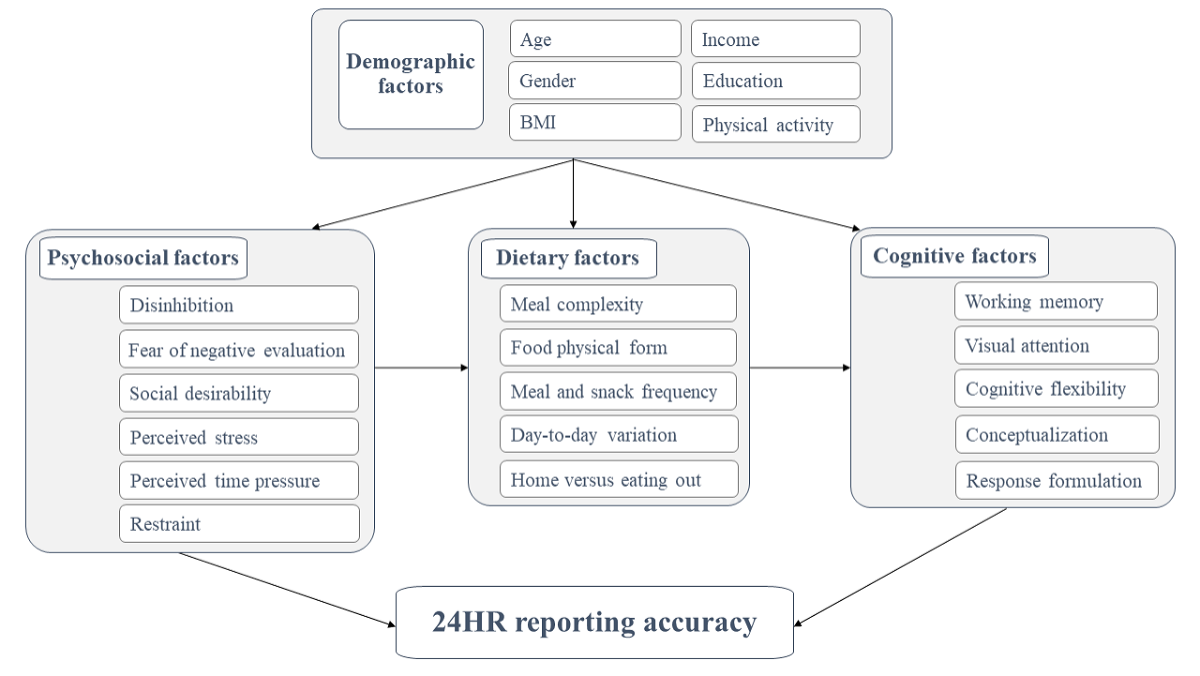
Conceptual framework for assessment of factors that may be associated with 24-hour dietary recall reporting accuracy. 24HR: 24-hour dietary recall.

Each participant will complete a web-based screening and provide informed consent. If eligible, they will be directed to complete web-based baseline demographic and psychosocial surveys (taking approximately 30 minutes) and cognitive tests (taking approximately 20 minutes) before attending 3 feeding days at the Curtin University School of Population Health food laboratory. Demographic characteristics, including age, gender, and highest education attainment will be recorded. Physical activity levels will be assessed using the International Physical Activity Questionnaire, short form 7-day self-administered format [49]. Table 1 presents a brief description of the questionnaires and the tests. Figure 2 shows a conceptual framework of the role of demographic, psychosocial, cognitive, and dietary factors in misreporting, synthesized from previous literature and adapted from existing frameworks [8,36,37,39,50-52].

**Table 1:** Baseline study questionnaire and test descriptions in Accuracy and Cost-effectiveness in Technology-Assisted Dietary Assessment.

Questionnaires and tests			Description of content		Study
Demographic and personal characteristics			11 items: gender, age, educational level, employment, country of birth, ethnicity, smoking, alcohol use, and socioeconomic and financial status		—^a^
Physical activity			8 items: International Physical Activity Questionnaire (short form)		[49]
**Psychosocial measures**					
	Three-factor eating	51 items measuring factors associated with eating behavior: cognitive restraint of eating, disinhibition, and hunger		[53,54]	
	Social desirability	13 items: true-false statements measuring social approval and acceptance		[55]	
	Fear of negative evaluation	12 items: 5-point scales to assess concern about being perceived unfavorably by others		[56]	
	Time pressure	7 items: assesses the competing perceived time pressure		[57]	
	Perceived Stress Scale	5 items: assesses perception of external environmental stressors; short version of the original Perceived Stress Scale-14		[58]	
	Weight loss history	2 items: assesses frequency and magnitude of previous weight loss.		[59]	
**Cognitive measures**					
	Visual digit span (forward and backward)	Assesses working memory by asking participants to recall spans of digits in 14 trials. Participants will see digit sequences on a computer or mobile phone screen and must recall them by selecting the recalled digits from a circle of digits with the mouse or their finger.		[60]	
	Vividness of visual imagery questionnaire	16 items: 5-point scale administered twice to assess conceptualization of visual memory. Participants imagine people and scenes and rate the vividness of these mental images (first with eyes open and then with eyes closed).		[61]	
	Trail making test	Assesses visual attention and task switching. Participants are asked to draw lines in specific, predetermined sequences from node to node on a screen as quickly and as accurately as possible.		[62]	
	Wisconsin card sorting test	Assesses cognitive flexibility and executive function. Participants are asked to sort cards into 4 different “categories.” No instructions are given regarding the categorization rules. Participants are informed whether each selection was correct or incorrect. The cards to sort into these piles have similar designs and vary in color (4 variants), shape (4 variants), and number of shapes (4 variants). Categorization rules change midtask without warning.		[63]	

^a^Question items devised by authors.

### Feeding Days

The participants will attend the food laboratory on 3 separate days. On day 1, they will receive a brief introduction to the study (with the objective described as finding better ways to assess what people eat) and be randomized. They will have their height and weight measured using standard protocols [64]. At the food laboratory, participants will consume 3 meals ad libitum (breakfast, lunch, and dinner) on the same day and will leave the laboratory between meals. No restrictions will be placed on consuming food and beverages outside of the laboratory meals. The participants will select from a menu and will not have access to the weight of their food and beverage selections. Menu items will be selected based on a combination of the top 100 most commonly consumed meals and snacks in Australia (Australian Bureau of Statistics, personal communication, 2020).

Each participant will enter the food laboratory one at a time to consume their meal. In accordance with the COVID-19 protocols issued by the Western Australia Government Department of Health, participants will be separated by screens and physically distanced from each other, with a maximum of 8 participants at any one time. This may change if restrictions are eased. All food and beverage items will be inconspicuously weighed using Kelba KHX-3 bench scales (Kelba) with a 0.1-gram resolution in a separate laboratory space before being served on the participant tray. Before delivering the tray to each participant, the researcher will inconspicuously take an image of the tray using a researcher version of the mFR24 app that allows insertion of a unique user ID for each image. When finished eating, each participant’s tray will be collected and an *after image* with the researcher mFR app will be taken. Plate waste will then be weighed to determine the amount of each item consumed. The amount consumed will be determined by subtracting the weight of the food plate waste from the weight of the served amount. Weighing will be conducted in duplicate, and a third measure will be taken if the first 2 measures differ by >0.5 grams. The average of the 2 closest measures will be recorded.

### 24-Hour Dietary Recall Interview Methods

Each day subsequent to the feeding day, the participants will complete a 24HR interview remotely, each time via a different technology-assisted dietary assessment method (ASA24, Intake24, or mFR24), the order of which will be randomized.

#### Automated Self-Administered Dietary Assessment Tool (2016)

Participants will be emailed a weblink and a username and password to access the ASA24 interface. A consortium of Australian Universities adapted the ASA24 by incorporating Australian food composition tables [65]. Participants will be asked to (1) report everything they had to eat and drink the previous day from midnight to midnight by selecting an eating occasion and time, then searching for matching food and beverage items, and reviewing any gaps in consumption of more hours; the database contains >4800 foods and beverages from Australian food composition tables (AUSNUT 2011-2013); (2) provide additional details of each food and beverage item (eg, form, preparation method, additions, and amount consumed); food images in ASA24 will assist in portion size estimation; (3) review and edit all the foods and beverages they selected; (4) add any commonly forgotten foods and beverages that they consumed after being prompted and directed back to the food list; and (5) confirm that they have recorded all of the food and beverages from the previous day [13]. Participants can add food and beverages that are not in the database via a *missing foods* tool incorporated in a bespoke spelling correction system to address misspelled food names. In previous studies, participants completed the ASA24 in 17 to 34 minutes [66].

#### Intake24

Participants will be emailed a weblink to access the Intake24 interface. A short instructional video is provided for the participants to watch before commencing the 24HR interview. Participants will be asked to (1) key in all the foods and beverages consumed the previous day between waking up and going to sleep as free text; (2) select items from a database and match each item consumed; the database contains >2800 foods and beverages by incorporating Australian food composition tables (AUSNUT 2011-2013) [67]; participants will be able to add food and beverages not included in the database via a *missing foods* tool incorporated in a bespoke spelling correction system to address misspelled food names; participants can add their own personal recipes, sandwiches, and salads; (3) estimate the portion sizes of the items consumed using images and standard serving sizes; (4) review all the foods and beverages they have selected and edit if necessary; and (5) add any missing items associated with the foods they have already selected after being prompted to do so [12]. Previous studies indicate an average completion time of 20 minutes.

#### Image-Assisted Mobile Food Record 24-Hour Recall

#### Overview

The mFR24 app, an image-based dietary assessment system [20,22,23,68], will be adapted as an image-assisted 24HR method for this study. The system consists of a mobile food record app (mFR24), which runs on a mobile device (iPhone or Android smartphone) and a dedicated cloud-based server for the storage of images, metadata, and food image processing and analysis. Using the mFR24, participants will take pictures of their food and beverages before and after eating. The images will be automatically uploaded to the cloud-based server via Wi-Fi or the 4G or 5G network. The content of the images will then be confirmed by the participant in a process known as user confirmation. A researcher version and a participant version of the app will be used in this study. The researcher version will be installed on various devices and used to take before and after images of each participant tray. These images will be uploaded to a researcher folder on the server. The participant version of the app will include additional features.

**Figure 3:**
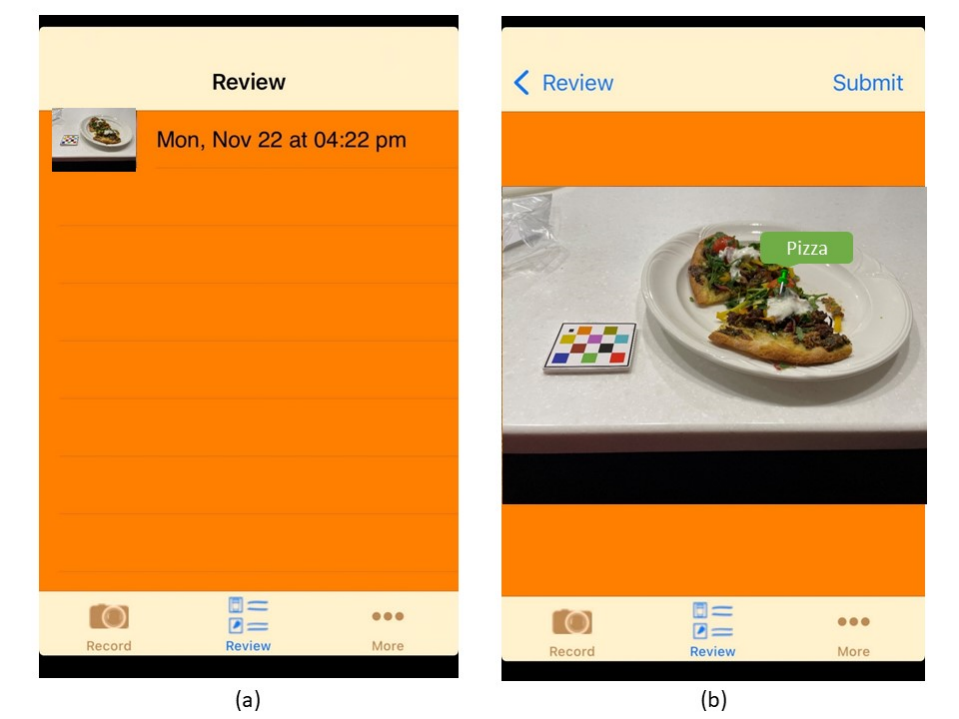
Screenshots of the mFR24 application interface showing the steps in the review process and viewing an eating occasion. (a) image list with time and date stamp displayed (b) viewing a labeled eating occasion with colored pins and labels identifying the food and beverages. The image also shows the inclusion of the fiducial marker.

When allocated to the mFR24, the participants will install the mFR24 (participant version) on their smartphones and be shown how to use the mFR24 app on the feeding day by a research assistant. They will be instructed to take a *before* and *after* image of all meals, beverages, and snacks consumed from the first meal served at the study center until midnight and include a fiducial marker (a colorful checked object of known size, color, and shape that assists in food and beverage recognition and quantification) in each image. mFR24 has an automated feature to detect the presence of the fiducial marker and alert participants if the fiducial marker is missing from the image. An angle-detection algorithm assists participants in taking the image at the correct angle (between 45° and 60° from the horizontal plane). Once captured and confirmed by the participant, the images will no longer be accessible to the participant until the user confirmation step (Figure 3). At the dinner session, participants will receive training on how to label their images for user confirmation the following day.

#### User Confirmation

The mFR24 app includes a feature known as the *user confirmation step*, where once midnight has lapsed, the images are returned to the participant for labeling with food and beverage names. On the day following the feeding day, participants will select and label each image from the review function in the mFR24 app (Figure 3). To label a food or beverage, the participant will tap on the item, and a pin will appear with *tap to edit*. Tapping the pin again will take the participant to the food list *search function*. When the participant starts typing the word, a list of foods will appear where they can select from the list. The food list consists of 372 food and beverage items. The food list has been adapted so that a *mini label* and *short description* are displayed to the participant. These labels link to a food composition database (not visible to the participant) with the food code, detailed description, and energy and nutrient composition (AUSNUT 2011-2013 nutrient database). If the participant cannot locate their food item in the list, typing *food not listed* will allow a free text entry. Once confirmed, the image with the confirmed pins is automatically sent to the server and disappears from the app. Participants will be asked to complete this task before the mFR24 interview. Researchers will be able to view the participant’s annotated images on a secure server.

#### mFR24 Interview

A trained researcher will conduct a 24HR interview following an adapted multiple-pass approach [69] outlined in Table 2. This 24HR process will enable the estimation of the total intake of food and beverages consumed during a 24-hour period. The recall process will be assisted by the labeled food and beverage images taken by the participant using the mFR24 app. The interview will be conducted via a video call on the day following the controlled feeding day. The interview structure is based on the AMPM developed by the United States Department of Agriculture [10]. Briefly, the original 5 steps include the collection of a quick list of foods and beverages consumed, a check for commonly forgotten foods, time and occasion, details and review, and a final probe. In the adapted version used in this study, the quick list and time pass will be completed by researchers before the interview using the participant labeled images, which will be viewed on a secure server.

**Table 2:** Adaptations of the United States Department of Agriculture Automated Multiple-Pass Method in the interview structure for the Image-Assisted mobile Food Record 24-Hour Recall interview.

Automated Multiple-Pass Method steps	Aim	Image-Assisted mobile Food Record 24-Hour Recall steps	
		When image is taken	When image is not taken
Step 1: quick list	The purpose of the quick list pass is to obtain a quick report of foods and beverages consumed in the past 24 hours without interrupting the respondent and to introduce the respondent to the concept of 24-hour recall.	Taken from the mini label and image provided by the participant; participant is asked to list any foods and beverages consumed that are not shown in images.	Participant is asked to list all foods and beverages consumed that are not shown in images.
Step 2: forgotten foods list and additions	The forgotten foods list prompts the respondent’s memory and collects other foods or beverages that are not reported in the quick list.	Participants are asked if they consumed items from a list of commonly forgotten foods.	Participants are asked if they consumed items from a list of commonly forgotten foods.
Step 3: time and occasion	The time and occasion of food or beverages consumed are recorded.	This does not require a separate pass. Time of eating is taken from the image metadata.	Ask participant to recall time and occasion of forgotten foods when item is reported.
Step 4: detail cycle	The aim of this step is to collect specific descriptive information about each food item and beverage reported and record quantities and any additions made to the food.	Clarify only nonidentifiable food and beverage items; follow the Australian Health Survey food model booklet to confirm amounts consumed; and check the after image for leftovers.	Use food-specific probes to obtain details; follow the Australian Health Survey food model booklet to confirm amounts consumed; and probe leftovers.
Step 5: final probe	This is the last opportunity for the respondent to remember any new foods and beverages.	Read out the list of food and beverage items.	Read out the list of food and beverage items.

The video call interview will consist of a quick list of any foods or beverages for which an image was not taken, a probe for commonly forgotten foods, a detail cycle, and a final probe. The researcher will use a screen-sharing function to enable both the researcher and participant to view each image simultaneously. During the detail cycle, participants will be asked to provide food and beverage details when these are unclear from the images. The participants will also be asked to describe the amount of each item consumed, using the standard food model booklet used in the Australian Health Survey [70]. Household measures such as metric teaspoons or pieces will also be used to describe amounts not available in the booklet and may, in some instances, be the preferred method of portion size description by the participants. During the final probe, the researcher will confirm all foods and beverages, descriptions, and portion sizes reported by the participant. The researcher conducting the 24HR interviews will not have access to the observed intake data and will not be present on the feeding day. Following the interview, data will be entered into a nutrition analysis software (FoodWorks 10, Xyris Software) linked to the AUSNUT 2011-2013 nutrient database to estimate food, energy, and nutrient intake for the 24-hour recall period.

#### Automated Image Analysis

Automated methods using computer vision and machine learning (eg, deep learning) techniques will also be undertaken [21-23] using the images collected with the mFR24. The study will test the accuracy of automated methods using computer vision and machine learning techniques to estimate true intake. These methods include food identification [71], food segmentation [72], and volume estimation (for food portion size estimation) [73]. In recent years, there has been rapid proliferation in the use of artificial intelligence and machine learning techniques in image analysis, particularly the use of deep learning methods based on neural networks [74]. The authors (EJD and FZ) from Purdue University have recently developed several deep neural network approaches for food image analysis and will modify these methods for use in this study. This will include generating ground truth images to train the deep neural networks using a series of standard images of the food and beverage items to be served at each meal with known food weights. As deep learning methods require a large amount of training data, we will investigate the use of data augmentation methods, particularly using generative adversarial networks [75], to aid in training. Trained models will then be used to recognize the food types, estimate the food portions, and compare the automated methods with the true intake. Standard metrics of precision and recall will be used in the computer vision and machine learning fields to determine the performance of the automated techniques.

### Poststudy Acceptability Questionnaire

The participant’s perceptions of the acceptability of the 24HR method will be asked at the end of the study via a web-based survey using both open and closed questions. Their perceptions of each method will be asked about one at a time in a randomized order. Participants will be asked what device they used to complete each method and then be asked questions using a 5-point Likert scale to rate their agreement with statements including how easy it was to find and remember foods and remember amounts and whether they would be willing to use the method again. Open-ended questions will explore the participant’s likes and dislikes about each method. Finally, participants will be asked which of the methods, if any, they preferred and why.

### Exit Interviews

A qualitative methodological approach using in-depth interviews and reflexive thematic analysis [76] will explore and describe perceptions and beliefs regarding each of the dietary assessment methods. Approximately 10% (20/150) of the recruited sample (participants stratified by age and gender) will be invited to participate in a semistructured in-depth interview. The script will include questions regarding what would motivate participants to take part in studies assessing dietary intake, barriers and enablers to participation, retention, and most-favored incentives to diet research participation (eg, dietary feedback and financial incentives). Questions will also be asked about the acceptability of the mFR24 method and features (eg, labeling of images). Interviews will be audio-recorded, transcribed verbatim, and reviewed for accuracy by the participant and researcher before analysis. Transcripts will be managed using transcription software (NVivo 12.6, QSR International). Sampling and analysis will continue until pragmatic saturation is reached [76].

### Cost and Cost-effectiveness of Measures

Both financial and time costs for participants to use each of the methods will be assessed. The time spent by researchers and participants in the administration of each 24HR method will be recorded for each participant. This includes time spent training individuals to take the mFR24 images, time spent completing the 24HR interview, and any time spent supporting individuals to complete their 24HR interviews. The time spent by researchers coding the mFR24 data will also be recorded. Both ASA24 and Intake24 have the capability to collect time stamp data to identify how much time the participants spend inputting data. All other time data, including time spent cleaning the data, will be manually collected by researchers.

Financial costs, such as server space for digital data storage, will be recorded. After converting the time data to a financial cost, the total cost data will be considered in parallel with the measures of accuracy derived for the 3 methods. The primary accuracy measure for cost-effectiveness is the absolute percentage error in energy intake. Comparing costs with accuracy will identify whether any of the 3 methods are dominated by another (ie, is more expensive and less accurate). If not, the cost per person to reduce absolute misreporting by 1% point will be estimated.

### Data Analyses

#### Energy Intake Estimation Accuracy

The participant feeding days with at least two meals eaten at the food laboratory will be included in the analyses. Daily energy intake from each 24HR method and the controlled feeding sessions will be calculated, excluding any items reportedly consumed outside of the food laboratory. Outlier checks will be conducted to identify any obvious keying errors or food composition data anomalies, which will be corrected before proceeding. Bland-Altman plots will be used to test for agreement between the 24HR values and controlled feeding measures at the individual and group levels. Repeated measurements will be analyzed using the linear mixed models procedure using SPSS version 25, accounting for age, gender, and BMI, with 24HR method and method order as fixed effects, to assess whether there are statistically significant differences by 24HR method. Misreporting will be assessed by examining the ratio of reported 24HR intake to observed intake, with the lowest tertile considered to indicate underreporting. The proportion of underreporters in each 24HR method will be compared using chi-square tests and logistic regression. Among underreporters, the total energy misestimation will also be compared using regression models adjusted for age, gender, and BMI.

#### Food Groups and Misreporting

Foods and beverages consumed during the controlled feeding sessions will be matched to the food codes from Australian food composition tables (AUSNUT 2011-2013) using a nutrition analysis software (FoodWorks 10, Xyris Software). The measured quantities of the foods and drinks consumed in the food laboratory will be coded. The observed intake of the provided food groups will be calculated. In ASA24, Intake24, and mFR24 data sets, foods and drinks that were reportedly consumed outside of the food laboratory (eg, snacks) will be removed before analysis based on the reported eating occasions and time.

The 24HR data on food and beverage intake from each method will be compared with the observed weight and daily intake of food and beverages to identify 4 types of misreporting, that is, omissions, intrusions, misclassifications, and portion misestimations. The following 5 steps will be used:

1. Matches between reported and observed intake will be identified by comparing the assigned food codes. Food codes correspond to a food grouping hierarchy in which the first 2 digits indicate the major food groups (eg, dairy, meat, and vegetables), the first 3 digits indicate the submajor food groups (eg, milk products and dishes), and the first 5 digits indicate the minor food groups (eg, dairy milk, yogurt, and cheese) [77]. Food codes of reported and observed intake data will be considered an exact match if they belong to the same minor food group. Foods codes from the same submajor food group will be considered a close match, whereas foods from the same major food group will be considered a far match.
2. Omissions, which are items that were consumed but are not reported, will be identified, and omission rates at each food group level will be calculated using the formula:
  sum of omissions / (sum of omissions + sum of all matches) × 100%.
  Omission rates of mixed meals, single items, and condiments will be calculated. Differences in omission rates by 24HR method will be assessed using chi-square tests.
3. The proportion of misclassifications, which are incorrect descriptions of consumed items, will be defined as close or far matches. Differences in the proportion of misclassifications within food groups by the 24HR method will be assessed using chi-square tests.
4. Intrusions, which are items that are reported but were not consumed, will be identified, counted by food group, and expressed in kilojoules.
5. Misestimation of portion size will be assessed by comparing the intake of food item matches in grams for each 24HR method with observed intake using a paired samples statistical test.

#### Correlates of Energy Misestimation

Psychosocial, demographic, and cognitive factors associated with omission and intrusion rates and with energy misestimation on each 24HR method will be evaluated using chi-square tests and multivariate logistic regression. The effects of factors will be reported as odds ratios and associated 95% CIs. Some dietary factors in the Accuracy and Cost-effectiveness of Technology-Assisted Dietary Assessment study are standardized because of the controlled feeding methodology (meal frequency and eating location), but it is hypothesized that food and meal complexity and physical form will affect reporting accuracy via an interaction with cognitive factors. P<.05 will be considered statistically significant in all analyses.

#### Acceptability

Differences in acceptability among methods indicated by the rating and proportions of participants agreeing with each of the ease-of-use statements will be assessed using chi-square tests. Demographic correlates of a preference for a particular method will be explored using multivariable logistic regression, adjusting for method order.

#### Cost-effectiveness

Both participant and researcher time costs will be multiplied by standard staff costs (including on-costs) to estimate the financial cost of using each method on a per-person basis. Differences in time cost and total financial cost will be compared across methods using a repeated measures analysis of variance test. P<.05 will be considered statistically significant. If statistically significant differences exist, differences in cost-effectiveness will be assessed using the same procedure. Cost-effectiveness will be defined as the cost per person to reduce the absolute misestimation of energy intake by 1% point.

## Results

Participant recruitment commenced in March 2021 and will end in December 2021. Ethics approval for this study was granted by the Institutional Review Board in April 2019. Participant recruitment commenced in March 2021. As of August 2021, 68 participants had enrolled in the study. Data collection will conclude at the end of December 2021. Data analysis will commence in 2022, and results are expected to be published in late 2022.

## Discussion

### Overview

This study will provide outcome results in 3 main areas. It will evaluate (1) the accuracy, user acceptability, and administration cost of 3 technology-assisted dietary assessment tools, which have never been compared in a single study; (2) the accuracy of novel automated image analysis technology; and (3) the association of reporting accuracy of participants with a range of cognitive and psychosocial factors.

The results of this research will provide additional information on the feasibility, accuracy, and cost to aid the selection and further development of 3 technology-assisted 24-hour recall methods for application in large-scale dietary assessment. The use of a controlled feeding study design for comparing multiple technology-based dietary assessment methods is novel and will allow comparison among methods relative to observed intake. The results will also elucidate the correlates of dietary intake misreporting, which will be useful in developing error mitigation strategies.

### Comparison With Previous Work

Measuring the cost of the 3 technology-assisted dietary assessment methods is a unique feature of this study. The costs of interviewer-administered pen and paper–based 24HR in 2013 ranged from US $178 per participant in South Asia to US $774 per participant in the Middle East and North Africa [78]. However, there is little published information on the operational costs of technology-assisted dietary assessment [79], although researchers have noted that staff costs persist despite substantial savings on data collection and entry [80]. Quantifying and comparing the costs of technology-assisted dietary assessment will provide essential information to aid decisions in planning population surveillance and large-scale epidemiological studies that aim to enroll thousands of individuals.

Elucidating the correlates of and developing methods for addressing misreporting in dietary data collection and analyses is relevant to the global research community involved in studies that assess dietary intake. It has been claimed that the measurement errors in dietary assessment are so great that the data hold no value [81,82]. A comprehensive refutation of this assertion argued that besides further developing and evaluating assessment methods, studies should be conducted to understand and manage measurement errors [29].

To date, studies evaluating the role of psychosocial factors in dietary reporting accuracy have focused on dietary energy misestimation, with energy expenditure as a reference measure [37,51,83-86]. Studies with measures of known food and beverage intake aid the understanding of how misreporting occurs, that is, the distributions of omissions, intrusions, incorrect portion sizes, and incorrect food descriptions [87-89]. However, to our knowledge, no such study has measured psychosocial and cognitive factors, and thus the associations with various food and beverage error types; this study aims to address this gap.

### Strengths and Limitations

This study has several strengths and limitations. A strength of this study is the collection of observed food and beverage intake using a controlled feeding study design. Many criterion validation studies use biomarkers of energy expenditure, which indicate the magnitude of misreporting, but do not help to understand differential misreporting of food and beverage items nor the underlying mechanisms. For example, certain food types may be more frequently omitted from reporting, or certain food portion sizes may be frequently misestimated. Another strength is the crossover design of the study, which allows for between- and within-group comparisons. Few studies have compared 24-hour recall methods with reference methods using a within-group comparison, and those studies did not use technology-based methods [90,91]. Studies assessing multiple technology-based 24HR methods have included different participants completing each method [33,46]. In such studies, between-person differences in dietary intake and recall biases may have contributed to the observed differences. The within-group comparison of the 3 dietary assessment methods in our study will enable a comparison not subject to confounding by between-person variation. This is an important strength of this study, as it will also allow the evaluation of consumer acceptability of these methods. The uniqueness of this study is the evaluation of the cost-effectiveness of the 24HR methods. This will provide valuable data for policy makers and researchers planning large-scale surveys.

A limitation of this protocol study is that the limited number of foods offered may facilitate a better chance of a match between reported and observed intakes than if the study was conducted in a free feeding environment. Another limitation of this study is the self-selecting sample, meaning that the findings may not be fully generalizable to the wider population, as study participants who volunteered may be more motivated to complete dietary assessment methods because of their own interest in dietary intake. Recruitment through the electoral roll is an important aspect of the study design that facilitates a wide and diverse recruitment. In addition, randomization by gender aims to recruit equal proportions of men and women across the groups so that gender differences can be assessed.

### Conclusions

The 24HR dietary assessment is a widely used method for population-wide nutrition surveillance and epidemiology globally [92]. By assessing the accuracy of dietary intakes, acceptability, and cost-effectiveness, this study will comprehensively evaluate 3 technology-assisted dietary assessment methods, which are administered remotely. The study will also determine if such methods can provide a cost-effective, efficient, and timely approach to large-scale data collection, which may translate to lower costs and improvements in scale, frequency of dietary intake surveillance, and better precision regarding food consumption.

## Abbreviations

24HR: 24-hour dietary recall
AMPM: Automated Multiple-Pass Method
ASA24: Automated Self-Administered Dietary Assessment Tool
mFR24: Image-Assisted mobile Food Record 24-Hour Recall

## References

[1] Byers T, Sedjo R, Willet W (2012). Nutrition monitoring and surveillance. Nutritional Epidemiology. 3rd Ed.

[2] (2014). 4363.0.55.001 - Australian Health Survey: Users' Guide, 2011-13. Australian Bureau of Statistics.

[3] (2019). AMPM - USDA automated multiple-pass method. Food Surveys Research Group, Agricultural Research Service, US Department of Agriculture.

[4] De Keyzer W, Bracke T, McNaughton S, Parnell W, Moshfegh A, Pereira R, Lee H, van't Veer P, De Henauw S, Huybrechts I (2015). Cross-continental comparison of national food consumption survey methods--a narrative review. Nutrients.

[5] Huybrechts I, Aglago EK, Mullee A, De Keyzer W, Leclercq C, Allemand P, Balcerzak A, Zotor FB, Gunter MJ (2017). Global comparison of national individual food consumption surveys as a basis for health research and integration in national health surveillance programmes. Proc Nutr Soc.

[6] Afshin A, Sur PJ, Fay KA, Cornaby L, Ferrara G, Salama JS, Mullany EC, Abate KH, Abbafati C, Abebe Z, Afarideh M, Aggarwal A, Agrawal S, Akinyemiju T, Alahdab F, Bacha U, Bachman VF, Badali H, Badawi A, Bensenor IM, Bernabe E, Biadgilign SKK, Biryukov SH, Cahill LE, Carrero JJ, Cercy KM, Dandona L, Dandona R, Dang AK, Degefa MG, El Sayed Zaki M, Esteghamati A, Esteghamati S, Fanzo J, Farinha CSES, Farvid MS, Farzadfar F, Feigin VL, Fernandes JC, Flor LS, Foigt NA, Forouzanfar MH, Ganji M, Geleijnse JM, Gillum RF, Goulart AC, Grosso G, Guessous I, Hamidi S, Hankey GJ, Harikrishnan S, Hassen HY, Hay SI, Hoang CL, Horino M, Ikeda N, Islami F, Jackson MD, James SL, Johansson L, Jonas JB, Kasaeian A, Khader YS, Khalil IA, Khang Y, Kimokoti RW, Kokubo Y, Kumar GA, Lallukka T, Lopez AD, Lorkowski S, Lotufo PA, Lozano R, Malekzadeh R, März W, Meier T, Melaku YA, Mendoza W, Mensink GB, Micha R, Miller TR, Mirarefin M, Mohan V, Mokdad AH, Mozaffarian D, Nagel G, Naghavi M, Nguyen CT, Nixon MR, Ong KL, Pereira DM, Poustchi H, Qorbani M, Rai RK, Razo-García C, Rehm CD, Rivera JA, Rodríguez-Ramírez S, Roshandel G, Roth GA, Sanabria J, Sánchez-Pimienta TG, Sartorius B, Schmidhuber J, Schutte AE, Sepanlou SG, Shin M, Sorensen RJ, Springmann M, Szponar L, Thorne-Lyman AL, Thrift AG, Touvier M, Tran BX, Tyrovolas S, Ukwaja KN, Ullah I, Uthman OA, Vaezghasemi M, Vasankari TJ, Vollset SE, Vos T, Vu GT, Vu LG, Weiderpass E, Werdecker A, Wijeratne T, Willett WC, Wu JH, Xu G, Yonemoto N, Yu C, Murray CJL (2019). Health effects of dietary risks in 195 countries, 1990–2017: a systematic analysis for the Global Burden of Disease Study 2017. The Lancet.

[7] Thompson F, Subar A, Coultson A, Boushey C, Ferruzzi M (2012). Dietary assessment methodology. Nutrition in the Prevention and Treatment of Disease.

[8] Nelson M, Atkinson M, Darbyshire S (2007). Food Photography I: the perception of food portion size from photographs. Br J Nutr.

[9] (2021). 24-hour Dietary Recall (24HR) At a Glance. National Institutes of Health National Cancer Institute.

[10] Blanton CA, Moshfegh AJ, Baer DJ, Kretsch MJ (2006). The USDA Automated Multiple-Pass Method accurately estimates group total energy and nutrient intake. J Nutr.

[11] Subar AF, Crafts J, Zimmerman TP, Wilson M, Mittl B, Islam NG, McNutt S, Potischman N, Buday R, Hull SG, Baranowski T, Guenther PM, Willis G, Tapia R, Thompson FE (2010). Assessment of the accuracy of portion size reports using computer-based food photographs aids in the development of an automated self-administered 24-hour recall. J Am Diet Assoc.

[12] Simpson E, Bradley J, Poliakov I, Jackson D, Olivier P, Adamson AJ, Foster E (2017). Iterative development of an online dietary recall tool: INTAKE24. Nutrients.

[13] Subar AF, Kirkpatrick SI, Mittl B, Zimmerman TP, Thompson FE, Bingley C, Willis G, Islam NG, Baranowski T, McNutt S, Potischman N (2012). The Automated Self-Administered 24-hour dietary recall (ASA24): a resource for researchers, clinicians, and educators from the National Cancer Institute. J Acad Nutr Diet.

[14] Bradley J, Simpson E, Poliakov I, Matthews J, Olivier P, Adamson A, Foster E (2016). Comparison of INTAKE24 (an Online 24-h Dietary Recall Tool) with interviewer-led 24-h recall in 11-24 Year-Old. Nutrients.

[15] Foster E, Lee C, Imamura F, Hollidge SE, Westgate KL, Venables MC, Poliakov I, Rowland MK, Osadchiy T, Bradley JC, Simpson EL, Adamson AJ, Olivier P, Wareham N, Forouhi NG, Brage S (2019). Validity and reliability of an online self-report 24-h dietary recall method (Intake24): a doubly labelled water study and repeated-measures analysis. J Nutr Sci.

[16] Park Y, Dodd K, Kipnis V, Thompson F, Potischman N, Schoeller D, Baer D, Midthune D, Troiano R, Bowles H, Subar A (2018). Comparison of self-reported dietary intakes from the Automated Self-Administered 24-h recall, 4-d food records, and food-frequency questionnaires against recovery biomarkers. Am J Clin Nutr.

[17] Boushey CJ, Spoden M, Zhu FM, Delp EJ, Kerr DA (2016). New mobile methods for dietary assessment: review of image-assisted and image-based dietary assessment methods. Proc Nutr Soc.

[18] Burrows TL, Ho YY, Rollo ME, Collins CE (2019). Validity of dietary assessment methods when compared to the method of doubly labeled water: a systematic review in adults. Front Endocrinol (Lausanne).

[19] Gemming L, Utter J, Ni Mhurchu C (2015). Image-assisted dietary assessment: a systematic review of the evidence. J Acad Nutr Diet.

[20] Zhu F, Bosch M, Woo I, Kim S, Boushey CJ, Ebert DS, Delp EJ (2010). The use of mobile devices in aiding dietary assessment and evaluation. IEEE J Sel Top Signal Process.

[21] He J, Shao Z, Wright J, Kerr D, Boushey C, Zhu F (2020). Multi-task image-based dietary assessment for food recognition and portion size estimation. Proceedings of the IEEE Conference on Multimedia Information Processing and Retrieval (MIPR).

[22] Zhu F, Bosch M, Khanna N, Boushey CJ, Delp EJ (2015). Multiple hypotheses image segmentation and classification with application to dietary assessment. IEEE J Biomed Health Inform.

[23] Wang Y, He Y, Boushey CJ, Zhu F, Delp EJ (2018). Context based image analysis with application in dietary assessment and evaluation. Multimed Tools Appl.

[24] Lazarte CE, Encinas ME, Alegre C, Granfeldt Y (2012). Validation of digital photographs, as a tool in 24-h recall, for the improvement of dietary assessment among rural populations in developing countries. Nutr J.

[25] Gemming L, Rush E, Maddison R, Doherty A, Gant N, Utter J, Ni Mhurchu C (2014). Wearable cameras can reduce dietary under-reporting: doubly labelled water validation of a camera-assisted 24 h recall. Br J Nutr.

[26] Hongu N, Pope BT, Bilgiç P, Orr BJ, Suzuki A, Kim AS, Merchant NC, Roe DJ (2015). Usability of a smartphone food picture app for assisting 24-hour dietary recall: a pilot study. Nutr Res Pract.

[27] Bulungu AL, Palla L, Priebe J, Forsythe L, Katic P, Varley G, Galinda BD, Sarah N, Nambooze J, Wellard K, Ferguson EL (2020). Validation of a life-logging wearable camera method and the 24-h diet recall method for assessing maternal and child dietary diversity. Br J Nutr.

[28] Arab L, Estrin D, Kim DH, Burke J, Goldman J (2011). Feasibility testing of an automated image-capture method to aid dietary recall. Eur J Clin Nutr.

[29] Subar A, Freedman L, Tooze J, Kirkpatrick S, Boushey C, Neuhouser M, Thompson F, Potischman N, Guenther P, Tarasuk V, Reedy J, Krebs-Smith S (2015). Addressing current criticism regarding the value of self-report dietary data. J Nutr.

[30] Freedman LS, Commins JM, Moler JE, Arab L, Baer DJ, Kipnis V, Midthune D, Moshfegh AJ, Neuhouser ML, Prentice RL, Schatzkin A, Spiegelman D, Subar AF, Tinker LF, Willett W (2014). Pooled results from 5 validation studies of dietary self-report instruments using recovery biomarkers for energy and protein intake. Am J Epidemiol.

[31] Rumpler WV, Kramer M, Rhodes DG, Moshfegh AJ, Paul DR (2008). Identifying sources of reporting error using measured food intake. Eur J Clin Nutr.

[32] De Keyzer W, Huybrechts I, De Maeyer M, Ocké M, Slimani N, van 't Veer P, De Henauw S (2010). Food photographs in nutritional surveillance: errors in portion size estimation using drawings of bread and photographs of margarine and beverages consumption. Br J Nutr.

[33] Kirkpatrick S, Subar A, Douglass D, Zimmerman T, Thompson F, Kahle L, George S, Dodd K, Potischman N (2014). Performance of the Automated Self-Administered 24-hour Recall relative to a measure of true intakes and to an interviewer-administered 24-h recall. Am J Clin Nutr.

[34] Lafrenière Jacynthe, Lamarche B, Laramée Catherine, Robitaille J, Lemieux S (2017). Validation of a newly automated web-based 24-hour dietary recall using fully controlled feeding studies. BMC Nutr.

[35] Widaman A, Keim N, Burnett D, Miller B, Witbracht M, Widaman K, Laugero K (2017). A potential tool for clinicians; evaluating a computer-led dietary assessment method in overweight and obese women during weight loss. Nutrients.

[36] Maurer J, Taren DL, Teixeira PJ, Thomson CA, Lohman TG, Going SB, Houtkooper LB (2006). The psychosocial and behavioral characteristics related to energy misreporting. Nutr Rev.

[37] Tooze J, Subar A, Thompson F, Troiano R, Schatzkin A, Kipnis V (2004). Psychosocial predictors of energy underreporting in a large doubly labeled water study. Am J Clin Nutr.

[38] Macdiarmid J, Blundell J (1998). Assessing dietary intake: who, what and why of under-reporting. Nutr Res Rev.

[39] Hébert JR (2016). Social desirability trait: biaser or driver of self-reported dietary intake?. J Acad Nutr Diet.

[40] Archer E, Shook RP, Thomas DM, Church TS, Katzmarzyk PT, Hébert JR, McIver KL, Hand GA, Lavie CJ, Blair SN (2013). 45-Year trends in women's use of time and household management energy expenditure. PLoS One.

[41] LeBlanc A, Gunnell K, Prince S, Saunders T, Barnes J, Chaput JP (2017). The ubiquity of the screen: an overview of the risks and benefits of screen time in our modern world. Translat J ACSM.

[42] (2015). General social survey: summary results, Australia, 2014. Australian Bureau of Statistics.

[43] Hutchesson MJ, Rollo ME, Callister R, Collins CE (2015). Self-monitoring of dietary intake by young women: online food records completed on computer or smartphone are as accurate as paper-based food records but more acceptable. J Acad Nutr Diet.

[44] Thompson FE, Dixit-Joshi S, Potischman N, Dodd KW, Kirkpatrick SI, Kushi LH, Alexander GL, Coleman LA, Zimmerman TP, Sundaram ME, Clancy HA, Groesbeck M, Douglass D, George SM, Schap TE, Subar AF (2015). Comparison of interviewer-administered and automated self-administered 24-hour dietary recalls in 3 diverse integrated health systems. Am J Epidemiol.

[45] Sharp DB, Allman-Farinelli M (2014). Feasibility and validity of mobile phones to assess dietary intake. Nutrition.

[46] Thompson FE, Dixit-Joshi S, Potischman N, Dodd KW, Kirkpatrick SI, Kushi LH, Alexander GL, Coleman LA, Zimmerman TP, Sundaram ME, Clancy HA, Groesbeck M, Douglass D, George SM, Schap TE, Subar AF (2015). Comparison of interviewer-administered and automated self-administered 24-hour dietary recalls in 3 diverse integrated health systems. Am J Epidemiol.

[47] Subar A, Ziegler R, Thompson F, Johnson C, Weissfeld J, Reding D, Kavounis K, Hayes R, Prostate‚ Lung‚ Colorectal‚Ovarian Cancer Screening Trial Investigators (2001). Is shorter always better? Relative importance of questionnaire length and cognitive ease on response rates and data quality for two dietary questionnaires. Am J Epidemiol.

[48] Rowland M, Adamson A, Poliakov I, Bradley J, Simpson E, Olivier P, Foster E (2018). Field testing of the use of INTAKE24-an online 24-hour dietary recall system. Nutrients.

[49] Craig CL, Marshall AL, Sjöström M, Bauman AE, Booth ML, Ainsworth BE, Pratt M, Ekelund U, Yngve A, Sallis JF, Oja P (2003). International physical activity questionnaire: 12-country reliability and validity. Med Sci Sports Exerc.

[50] Krall EA, Dwyer JT, Ann Coleman K (1988). Factors influencing accuracy of dietary recall. Nutri Res.

[51] Karelis AD, Lavoie M, Fontaine J, Messier V, Strychar I, Rabasa-Lhoret R, Doucet E (2010). Anthropometric, metabolic, dietary and psychosocial profiles of underreporters of energy intake: a doubly labeled water study among overweight/obese postmenopausal women--a Montreal Ottawa New Emerging Team study. Eur J Clin Nutr.

[52] Szenczi-Cseh J, Horváth Z, Ambrus A (2017). Validation of a food quantification picture book and portion sizes estimation applying perception and memory methods. Int J Food Sci Nutr.

[53] Stunkard AJ, Messick S (1985). The three-factor eating questionnaire to measure dietary restraint, disinhibition and hunger. J Psychosom Res.

[54] Bond MJ, McDowell AJ, Wilkinson JY (2001). The measurement of dietary restraint, disinhibition and hunger: an examination of the factor structure of the Three Factor Eating Questionnaire (TFEQ). Int J Obes Relat Metab Disord.

[55] Reynolds WM (1982). Development of reliable and valid short forms of the marlowe-crowne social desirability scale. J Clin Psychol.

[56] Leary MR (2016). A brief version of the Fear of Negative Evaluation Scale. Pers Soc Psychol Bull.

[57] van der Lippe T (2016). Dutch workers and time pressure: household and workplace characteristics. Work Employ Soc.

[58] Santiago PH, Nielsen T, Smithers LG, Roberts R, Jamieson L (2020). Measuring stress in Australia: validation of the perceived stress scale (PSS-14) in a national sample. Health Qual Life Outcomes.

[59] Myers VH, McVay MA, Champagne CM, Hollis JF, Coughlin JW, Funk KL, Gullion CM, Jerome GJ, Loria CM, Samuel-Hodge CD, Stevens VJ, Svetkey LP, Brantley PJ (2013). Weight loss history as a predictor of weight loss: results from Phase I of the weight loss maintenance trial. J Behav Med.

[60] Woods DL, Kishiyamaa MM, Lund EW, Herron TJ, Edwards B, Poliva O, Hink RF, Reed B (2011). Improving digit span assessment of short-term verbal memory. J Clin Exp Neuropsychol.

[61] Marks D (1973). Visual imagery differences in the recall of pictures. Br J Psychol.

[62] Reitan RM (2016). Validity of the trail making test as an indicator of organic brain damage. Percept Mot Skills.

[63] Nelson HE (1976). A modified card sorting test sensitive to frontal lobe defects. Cortex.

[64] Stewart A, Marfell-Jones M, Olds T, de Ridder J (2011). International Standards for Anthropometric Assessment.

[65] (2020). ASA24 - Australia. National Institutes of Health National Cancer Institute.

[66] (2020). ASA24® Frequently Asked Questions (FAQs). National Institutes of Health National Cancer Institute.

[67] (2019). Localisation and internationalisation. Intake24 - Food Standards Scotland, Newcastle University, University of Cambridge, Monash University.

[68] Ahmad Z, Kerr DA, Bosch M, Boushey CJ, Delp EJ, Khanna N, Zhu F (2016). A mobile food record for integrated dietary assessment. MADiMa16 (2016).

[69] Moshfegh AJ, Rhodes DG, Baer DJ, Murayi T, Clemens JC, Rumpler WV, Paul DR, Sebastian RS, Kuczynski KJ, Ingwersen LA, Staples RC, Cleveland LE (2008). The US Department of Agriculture Automated Multiple-Pass Method reduces bias in the collection of energy intakes. Am J Clin Nutr.

[70] (2010). Australian Health Survey: Food model booklet. Australian Bureau of Statistics.

[71] Mao R, He J, Shao Z, Yarlagadda S, Zhu F (2021). Visual aware hierarchy based food recognition. Pattern Recognition.

[72] Wang Y, Zhu F, Boushey C, Delp E (2017). Weakly supervised food image segmentation using class activation maps. Proceedings of the IEEE International Conference on Image Processing (ICIP).

[73] Fang S, Zhu F, Jiang C, Zhang S, Boushey C, Delp E (2016). A comparison of food portion size estimation using geometric models and depth images. Proceedings of the IEEE International Conference on Image Processing (ICIP).

[74] LeCun Y, Bengio Y, Hinton G (2015). Deep learning. Nature.

[75] Goodfellow I, Pouget-Abadie J, Mirza M, Xu B, Warde-Farley D, Ozair S, Courville A, Bengio Y (2020). Generative adversarial networks. Commun ACM.

[76] Braun V, Clarke V (2019). Reflecting on reflexive thematic analysis. Qual Res Sport Exerc Health.

[77] (2019). Classification of foods and dietary supplements. Food Standards Australia & New Zealand.

[78] Fiedler JL, Martin-Prével Y, Moursi M (2013). Relative costs of 24-hour recall and Household Consumption and Expenditures Surveys for nutrition analysis. Food Nutr Bull.

[79] Amoutzopoulos B, Steer T, Roberts C, Cade JE, Boushey CJ, Collins CE, Trolle E, de Boer EJ, Ziauddeen N, van Rossum C, Buurma E, Coyle D, Page P (2018). Traditional methods new technologies - dilemmas for dietary assessment in large-scale nutrition surveys and studies: a report following an international panel discussion at the 9th International Conference on Diet and Activity Methods (ICDAM9), Brisbane, 3 September 2015. J Nutr Sci.

[80] Kirkpatrick S, Gilsing A, Hobin E, Solbak N, Wallace A, Haines J, Mayhew A, Orr S, Raina P, Robson P, Sacco J, Whelan H (2017). Lessons from studies to evaluate an online 24-hour recall for use with children and adults in Canada. Nutrients.

[81] Archer E, Hand GA, Blair SN (2013). Validity of U.S. nutritional surveillance: national health and nutrition examination survey caloric energy intake data. PLoS One.

[82] Archer E, Pavela G, Lavie CJ (2015). The inadmissibility of what we eat in America and NHANES Dietary Data in Nutrition and Obesity Research and the Scientific Formulation of National Dietary Guidelines. Mayo Clin Proc.

[83] Hebert J (2002). Systematic errors in middle-aged women's estimates of energy intake comparing three self-report measures to total energy expenditure from doubly labeled water. Ann Epidemiol.

[84] Novotny JA, Rumpler WV, Riddick H, Hebert JR, Rhodes D, Judd JT, Baer DJ, McDowell M, Briefel R (2003). Personality characteristics as predictors of underreporting of energy intake on 24-hour dietary recall interviews. J Am Diet Assoc.

[85] Mossavar-Rahmani Y, Tinker LF, Huang Y, Neuhouser ML, McCann SE, Seguin RA, Vitolins MZ, Curb JD, Prentice RL (2013). Factors relating to eating style, social desirability, body image and eating meals at home increase the precision of calibration equations correcting self-report measures of diet using recovery biomarkers: findings from the Women's Health Initiative. Nutr J.

[86] Taren DL, Tobar M, Hill A, Howell W, Shisslak C, Bell I, Ritenbaugh C (1999). The association of energy intake bias with psychological scores of women. Eur J Clin Nutr.

[87] Kirkpatrick S, Subar A, Douglass D, Zimmerman T, Thompson F, Kahle L, George S, Dodd K, Potischman N (2014). Performance of the Automated Self-Administered 24-hour Recall relative to a measure of true intakes and to an interviewer-administered 24-h recall. Am J Clin Nutr.

[88] Lafrenière J, Lamarche B, Laramée C, Robitaille J, Lemieux S (2017). Validation of a newly automated web-based 24-hour dietary recall using fully controlled feeding studies. BMC Nutr.

[89] Rumpler WV, Kramer M, Rhodes DG, Moshfegh AJ, Paul DR (2008). Identifying sources of reporting error using measured food intake. Eur J Clin Nutr.

[90] Bingham SA, Gill C, Welch A, Day K, Cassidy A, Khaw KT, Sneyd MJ, Key TJ, Roe L, Day NE (1994). Comparison of dietary assessment methods in nutritional epidemiology: weighed records v. 24 h recalls, food-frequency questionnaires and estimated-diet records. Br J Nutr.

[91] Tran KM, Johnson RK, Soultanakis RP, Matthews DE (2000). In-person vs telephone-administered multiple-pass 24-hour recalls in women. J Am Dietet Assoc.

[92] Baranowski T, Willett W (2012). 24-hour recall and diet record methods. Nutritional Epidemiology, 3rd Ed.

[93] (2004). Apache Licence, Version 2. The Apache Software Foundation.

